# Impact assessment of the medical practice assisting (MPA) program in general practice in the hunter New England and central coast regions of Australia

**DOI:** 10.1186/s12960-022-00781-6

**Published:** 2022-12-05

**Authors:** Shanthi Ann Ramanathan, Rod Ling, Alison Tattersall, Nicola Ingold, Mary Sheffi De Silva, Shara Close, Andrew Searles

**Affiliations:** 1 Hunter Medical Research Institute, Newcastle, Australia; 2 University of Newcastle College of Health, Medicine and Wellbeing, Newcastle, Australia; 3 Hunter New England Central Coast Primary Health Network, Newcastle, Australia

**Keywords:** Impact assessment, Rural health workforce, Medical practice assisting, Workforce retention, Regional, Remote, Education pathways

## Abstract

**Background:**

A regional Australian Primary Health Network (PHN) has been subsidising administrative staff from local general practices to undertake the Medical Practice Assisting (MPA) course as part of its MPA Program. The MPA Program aimed to upskill administrative staff to undertake clinical tasks and fill in for busy or absent Practice Nurses (PNs), freeing up PNs to increase revenue-generating activity, avoiding casual replacement staff wages, and increasing patient throughput. An impact assessment was undertaken to evaluate the impact and estimate the economic costs of the MPA program to the PHN, general practices, and students to inform future uptake of the intervention.

**Methods:**

The Framework to Assess the Impact of Translational Health Research (FAIT) was utilised. Originally designed to assess the impact of health research, this was its first application to a health services project. FAIT combines three validated methods of impact assessment—Payback, economic analysis and narratives underpinned by a program logic model. Quantified metrics describe the impacts of the program within various “domains of benefit”, the economic model costs the intervention and monetises potential consequences, and the narrative tells the story of the MPA Program and the difference it has made. Data were collected via online surveys from general practitioners (GPs), PNs, practice managers; MPA graduates and PHN staff were interviewed by phone and on Zoom.

**Results:**

FAIT was effective in evidencing the impacts and economic viability of the MPA Program. GPs and PNs reported greater work satisfaction, PNs reported less stress and reduced workloads and MPA graduates reported higher job satisfaction and greater confidence performing a range of clinical skills. MPA Program economic costs for general practices during candidature, and 12 month post-graduation was estimated at $69,756. With effective re-integration planning, this investment was recoverable within 12 months through increased revenue for practices. Graduates paid appropriately for their new skills also recouped their investment within 24 months.

**Conclusion:**

Utilisation of MPA graduates varied substantially between practices and COVID-19 impacted on their utilisation. More strategic reintegration of the MPA graduate back into the practice to most effectively utilise their new skillset could optimise potential benefits realised by participating practices.

**Supplementary Information:**

The online version contains supplementary material available at 10.1186/s12960-022-00781-6.

## Background

In Australia, primary health care (PHC) covers health care unrelated to a hospital visit including health promotion, prevention, early intervention, treatment of some acute conditions, and management of chronic conditions. Through a fee-for service arrangement administered by the Australian Federal government, primary health care services are delivered by community health centres, Aboriginal community-managed medical services and allied health clinics, but the bulk of PHC is provided by general practices, the setting of focus in this study. These practices have a mix of staffing including general practitioners (GPs), practice nurses (PNs), practice managers (PMs), allied health professionals and administrative staff/receptionists.

Workforce issues in general practice, mainly the recruitment and retention of GPs and PNs, have been concerning across many parts of Australia [1–5] and other developed nations [6–8], particularly in rural and remote practices where there is added complexity [1, 7–9]. These workforce issues have implications for access and quality of care provided to residents in these regions and ultimately to the ongoing management of their health. In Australia, about 28% of the population live in rural and remote areas [10]. In comparison to their urban counterparts, these Australians have poorer access to, and use of, primary health care services including general practice, and also have poorer health outcomes, higher rates of injury, hospitalisations, and deaths [10].

A range of policy, legislative and industry initiatives have been utilised to address these workforce issues such as the Stronger Rural Health Strategy and the National Strategic Framework for Rural and Remote Health [1, 5, 11, 12]. Solutions have focussed primarily on offering financial and visa incentives to entice both local and overseas-trained general practice staff to relocate to rural and remote locations. Research indicates that these initiatives have been less successful than those focused on training local personnel, improving the productivity and job satisfaction of practice staff, and improving efficiencies to optimise time spent with patients [13]. One such strategy is to extend the scope of practice for existing health professionals and other general practice staff [3, 14, 15]. These initiatives are rarely evaluated [2, 5, 6]. Evaluations that measure meaningful impacts of interventions on key implementers and beneficiaries, and the cost and expected returns from investment, are vital for ensuring resources are not wasted on initiatives that are ineffective [2, 5, 6, 12, 15–17].

In Australia, primary health networks (PHNs) were established by the Federal Government to increase efficiency and effectiveness of medical services and improve coordination of care to ensure patients receive the right care, in the right place, at the right time [18]. In 2018, the Hunter New England and Central Coast Primary Health Network (HNECC PHN hereafter also referred to as PHN), began an initiative—subsidising administrative staff from local general practices and Aboriginal Medical Services (AMS) to undertake a Certificate IV in Medical Practice Assisting (hereafter referred to as the MPA Program) offered by UNE Partnerships, a registered training organisation associated with the University of New England (UNE). The aim of the MPA Program was to upskill administrative staff in general practices (whose main duties included tasks such as answering the phone, booking appointments and ordering supplies) to undertake selected clinical tasks and higher order administrative tasks (see list of these in Additional file 4). This would free up PNs and GPs to work at the top of their scope, thereby increasing productivity and throughput within general practices [16], improving patient care and experience, and contributing to workforce retention in primary care [19].

The MPA Program originated in 2003 at GP Partners, a division of general practice in Queensland [20]. In 2007 the Certificate IV in Medical Practice Assisting was included in the national health training package and endorsed by all Australian states and territories [21]. However, a formal evaluation of the impact from the MPA program has never been conducted.

In 2020, the Embedded Economist program funded by New South Wales Regional Health Partners and the Australian Medical Research Future Fund was implemented at HNECC PHN. The program enabled PHN staff to work with health economists and impact specialists from the Hunter Medical Research Institute to undertake an impact evaluation of the MPA Program, as it was being applied by the PHN. It was anticipated that the results would inform ongoing PHN investment in the MPA program and inform general practices and their staff considering this intervention in the future, not just in Australia, but in other developed nations facing similar workforce issues.

The primary aims of the impact evaluation were to assess the impact of the MPA Program on participating general practices and their staff, capture the economic cost of the MPA Program to the PHN, practices and students, and determine whether the monetisable consequences^1^ of the investment represented a good return on the investment. A secondary aim was to apply the Framework to Assess the Impact of Translational Health Research (FAIT) methodology to a non-research program of work and assess its suitability in effectively evaluating the impacts of a health services led workforce development program.

## Methods

### Setting

The setting for the impact assessment was the HNECC PHN and general practices across the Hunter, New England, and Central Coast regions in the Australian state of New South Wales who had participated in the MPA Program in 2018 and 2019. The HNECC regions cover an area of approximately 130 000 square kilometres and are home to 1.2 million residents, a quarter of whom live in rural, regional, and remote locations. The combined area has a total of 410 general practices and nine Aboriginal Medical Services; 65 of them participated in the MPA Program in 2018 and 2019 [22].

### Evaluation design

FAIT was developed specifically to improve research translation and to optimise and assess the impact from research investments [23]. FAIT has been demonstrated to be an effective impact assessment tool in a range of Health and Medical Research (HMR) projects in Australia and internationally [24, 25]. It combines three validated methods of impact assessment (Payback, Economic analysis, and Narratives) to present a multidimensional, comprehensive approach to assessing the impact of research projects and programs. A detailed description of the FAIT method can be found in Additional file 1. This study represents the first application of FAIT to a non-research program of work.

#### Program logic model and payback

**Fig. 1:**
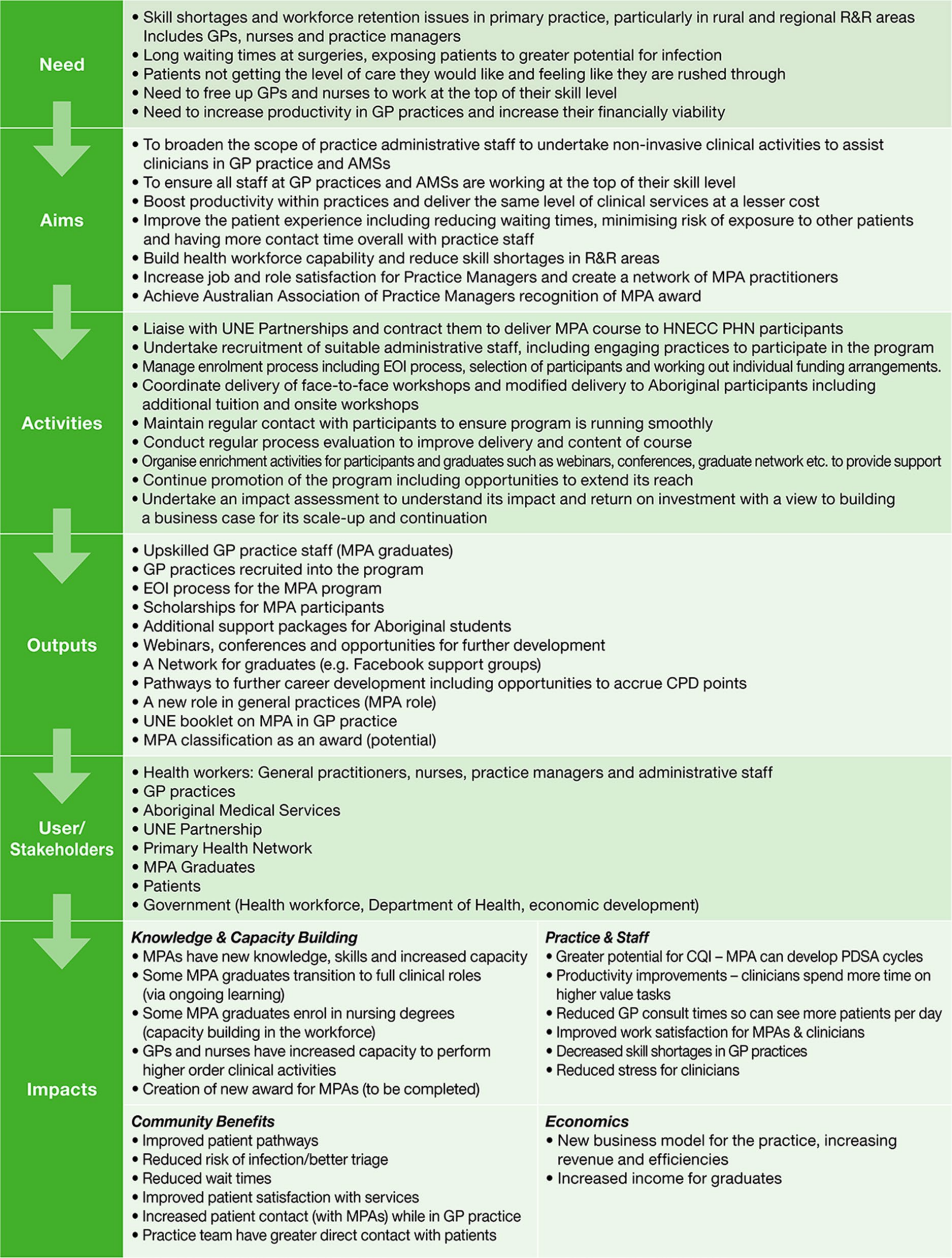
Impact logic model and scorecard

A detailed program logic model (PLM) was developed to map the pathway between the need for the MPA Program and the eventual impact of the intervention (see Fig. 1). Within FAIT, the PLM underpins all three methods.

Next, a modification of the Payback Framework, first developed by Buxton and Hanney [26], was applied. The Payback Framework captures impact within broad domains of benefit. For the MPA project the relevant domains were selected by the evaluators (SR, RL, AS & SD) in collaboration with PHN staff (AT, NI) to reflect the anticipated benefits of the MPA program from the various perspectives. The selected domains were: knowledge advancement, capacity building, practice change, community benefits and economic impacts. Existing metrics were selected or specifically developed to capture the intended impacts of the MPA Program such as skills development, job satisfaction and workforce productivity.

#### Economic evaluation

A cost-consequence analysis (CCA) was used to compare costs of the MPA program to its potential consequences for general practices and for MPA students. A CCA does not limit itself to standard metrics like cost–benefit ratios. Rather it lays out the costs and consequences for decision makers to make their own subjective judgements on program cost-efficacy and whether the investment choice represents value for money.

### Costs

All resources used for the MPA program were reported in monetary terms (economic costs). This included actual cash costs and the ‘opportunity costs’ of directing resources to the MPA program rather than another activity. A bottom-up approach was used to capture all resources from the perspectives of HNECC PHN, participating practices and MPA students. All resources were identified, valued, and aggregated separately as detailed in Additional file 2. Costs are presented in monetary units so that the value of different resources can be aggregated and compared. Unit costs and cost assumptions are captured in Additional files 2 and 3, and in Table 3.

### Consequences

Consequences were focussed on realised and potential benefits for general practices and MPA graduates. These would also indirectly benefit the PHN whose main aim and function is to improve the delivery and quality of primary care, including general practice, within its jurisdiction. The consequences included were limited to those that were monetisable. Consequences not readily monetisable (e.g., job satisfaction and increased confidence in performing clinical tasks) are presented in their natural units and discussed within the Payback metrics (Table 2).

#### General practices

The main monetisable consequences were efficiencies gained through utilisation of MPA graduates in their upskilled role for half a day, every day; and opportunities for the practice to gain from increases in billable services. The administrative duties performed by the MPA graduate prior to the completion of their course (i.e. undertaking general reception and administrative duties) would be picked up by the Practice Manager or a casual administration assistant. Financial and administrative data to inform on actual level of billable services was unavailable, so modelling was informed by survey data and supplemented with expert input from PHN staff.

#### MPA graduates

Monetizable consequences for graduates were their potential higher remuneration following course completion. Additional wages were projected over a full year and based on survey responses and an aspirational goal of MPAs being paid $3.00 extra per hour (mid-point between the receptionist and enrolled nurse wage scale). Sensitivity analyses was used to provide the range of potential returns to both general practices and MPA graduates.

#### Narrative

The narrative was built from the program logic model and impacts and responses from study participants, all of whom provided consent for their qualitative responses to be included in the analysis. Comments were grouped by key themes and captured the various perspectives around impact. The results for the application of FAIT to the MPA Program are summarised and presented in a scorecard format by each method.

#### Data collection

Data collection methods for each component of FAIT are summarised here:

*Payback*: Online surveys and interviews with participants and administrative records from the HNECC PHN.

*Economics*: Online surveys and interviews with participants, administrative records from the HNECC PHN, and secondary data and expert input provided by the MPA Coordinator and Primary Care Improvement Officer from the PHN.

*Narrative*: Online surveys and interviews with participants.

#### Participants

Participants represented five groups of general practice staff: (1) practice managers; (2) current MPA students; (3) PN’s; (4) GP’s and (5) MPA graduates. Eligible general practices were those that were listed as having at least one MPA student enrolled between January 2018 and December 2019. Eligible practice managers, PNs and GPs were those who were working at those practices and eligible MPA students were those who were currently enrolled in the MPA Program, at the time of the survey. Eligible MPA graduates were those who had graduated by June 2020. All eligible practices were invited to participate. Of the 62 eligible practices, 36 had one current MPA student (58%) and 26 (42%) had a returned graduate. Of these 26 practices, 4 had their MPA graduate leave their practice by the time of the survey, leaving only 22 eligible MPA graduates. To supplement general practice staff views, the MPA Coordinator and Primary Care Improvement Officer from the PHN, and the Program Manager for Health Programs at UNE Partnerships were consulted due to their experience with the program and capacity to provide contextual clarity.

#### Surveys and interviews

Online surveys were programmed and deployed using REDCap (Research Electronic Data Capture) a secure web application for building and managing online surveys and databases [28]. Five surveys were developed, one for each participant group. All groups, except for MPA graduates, self-administered the survey. Graduate surveys were administered via telephone interview with a member of the HMRI team who entered the graduate’s answers for them. This was to enable the capture of more detailed responses with regards to the impact of the program on graduates personally. A pragmatic pilot involving two practices from the cohort was undertaken. Suggested changes were incorporated prior to deployment.

## Results

A total of 57 general practice staff completed surveys (see Table 1 for details of response rates). The MPA Coordinator and Primary Care Improvement Officer from the HNECC PHN provided supplementary information. Results are presented by the three FAIT methods.

**Table 1:** MPA survey: participants by role

Participant role	Completed surveys	% if taking all practices into account (n=62)	% If only taking practices with a current graduate (n=22)
Practice manager	19	30.6	–
MPA graduate	11		50
MPA student	13	21	–
GP	5	8	–
Nurse practitioner	9	14.5	–
Total	57		

### Payback

Table 2 reports on the impacts of the MPA Program by way of impact metrics, grouped within five domains of impact—knowledge advancement, capacity building, practice change, community benefit and economic benefit. Key impacts included the proportion of MPA graduates with increased confidence to perform clinical tasks, the proportions performing various clinical tasks post-graduation, their increase in job satisfaction and the increased throughput of patients through general practices.

**Table 2:** Payback metrics

Domains of benefit	Categories of impact	Results
Knowledge advancement	Students Presentations Podcast	108 Students have been exposed to the MPA Program and knowledge about clinical aspects of a general practice 2 Presentations (one at MPA Conference and one to Federal Regional Health Minister Mark Coulton) 581 Views
	MPA Conference	64 Participants 15 Presentations
	Online reporting	https://nswregionalhealthpartners.org.au/2020/12/17/nsw-regional-health-partners-welcomes-regional-health-minister-mark-coulton/
Capacity building	Graduates Practices Aboriginal Medical Services Rural/Regional locations	44 Graduates by 1 June 2021 63 Practices with at least 1 student commencing the program in 2018 or 2019 22 Practices with returned MPA graduates by October 2020 2 AMS with an MPA student 30 General practices in rural and regional locations (48% of practices)
	Indigenous graduates	2 Indigenous graduates
	Currently working	22 MPA graduates working in October 2020
	Further education	4 Graduates currently enrolled in further study as a result of completing the MPA Program
	Confidence to perform skills	Since graduation from the program, 100% of MPA graduates reported increased confidence in: • Handling specimens for onsite testing, • Responding to adverse events, • Measuring height and weight, • Measuring vital signs, • Measuring blood glucose, • Conducting an electrocardiogram, • Conducting colour blindness, visual acuity, audiometry and spirometry testing and providing assistance in the provision of care 91% reported increased confidence in: • Processing reusable medical devices, • Conducting pregnancy testing, • Confirming physical health status • Providing advanced first aid management 82% reported increased confidence in: • Triaging patients, • Complying with infection control Between 63 and 72% reported increased confidence in: • Supporting quality improvement initiatives • Maintaining medication stocks
Practice change	Proportion of MPAs performing these tasks since graduating (who were not performing them before)	100% reported • Measuring vital signs, • Complying with infection control • Maintaining patient records and information systems 91% reported • Measuring blood glucose, • Doing visual acuity, audiometry and spirometry testing 82% reported • Measuring height and weight, • Conducting electrocardiography, • Assisting with delivery of care • Processing reusable medical devices 72% reported • Providing advanced first aid • Confirming physical health status • Pregnancy testing • Maintaining medication and immunisation stocks
	Proportion who are performing these tasks who are competent	•100% reported performing non- clinical tasks like supporting CQI processes, triaging patients, processing accounts, maintaining patient records and information systems, complying with infection control and clinical tasks like measuring vital signs •91% are handling specimens for offsite testing, responding to adverse events, maintaining medication/immunisation stocks, measuring height and weight, blood glucose and testing to colour blindness, visual acuity, audiometry and spirometry
	Practice nurses reported spending less time on the following tasks	•71% reported spending less time handling specimens for offsite testing •42% spent less time on non-clinical tasks like maintaining patient records, supporting CQI processes and preparing accounts •37.5% spent less time on measuring vital signs, height, weight and blood glucose, pregnancy testing, conducting ECGs, assisting the GP in the delivery of care, processing reusable devices and triaging patients
	Improved throughput/increased productivity since MPA graduated	•1 GP reported the practice being able to see more patients for blood tests, international normalised ratio (INR) for monitoring of warfarin and electrocardiograms •3 Practice Managers reported their practice seeing between 5 and 12 extra patients per day and the Practice Nurse seeing 2–8 extra patients per day •4 Practice Managers reported an improvement in triaging practice including better medical histories, skills and confidence •3 Practice Managers reported an improvement in CQI processes resulting in better overall work practices, use of RN time and patient satisfaction
Community benefit	MPA Graduates	•100% Reported personal and/or professional benefits as a result of the MPA Program •82% Reported an increase in their job satisfaction since completion •90% Reported a positive impact on their practice •45% Have gone on to do further study •36% Have seen a wage increase
	Practice Nurses	•43% Reported greater work satisfaction •43% Reported less stress at work
	General practitioners	•3 GPs claimed to have increased work satisfaction since MPA Graduate completed their course •1 GP claimed reduced stress
	Patients	•3 Practice Managers reported an improvement in waiting times (by 50% and at the Diabetes Clinic) •2 Practice Managers had evidence of greater patient satisfaction (received positive feedback from patient and from the patients of the Diabetic Clinic) •1 GP reported improvement in patient care and more resources devoted to patient care and observations
Economic benefit	Increased revenue	•3 Practice Nurses claimed a 10% increase in billable items once the MPA had returned and 1 claimed a 5% increase •3 Practice Managers reported their practice seeing between 5 and 12 extra patients per day and the Practice Nurse seeing 2–8 extra patients per day
	Increased earnings for MPA graduates	•4 MPA’s reported increased earnings of between $1.50 and $3.00 per hour totalling between $2974 and $5948 per year (7 reported no increase in wages)

### Cost-consequence analysis

#### Costs

In 2018, when 44 students commenced and received scholarships, the total annual economic cost to the PHN for the MPA Program was $126 718, the largest item being $94 600 for scholarships (see Table 3 (1)). General practice costs (see Table 3 (2)) were separated by (a) costs during the candidature and (b) costs post candidature. Total economic cost during candidature is estimated at $22 687. During the candidature period, main costs were labour during on-the job training time, (MPA student who is backfilled by a practice manager and/or a casual administrative assistant) ($15 401) and student study leave ($3271). The total economic costs for 12 months post-graduation was estimated at $47 070; and the total economic cost for both periods was $69 756 per MPA graduate, including increased wages for the graduate with on-costs. For students, total economic costs for the 18-month course per individual was estimated at $3729 (Table 3 (3)) including personal computers, stationery, and travel to face-to-face workshops.

**Table 3:** Cost and monetary consequences of the MPA Program

Costs				
(1) Hunter New England and Central Coast Primary Health Network (PHN)—Annual Economic costs^a^			Item cost ($AUD)	Total ($AUD)
Labour	Team leader	4.5 h/week	$10 957	
	Web Support/comms	7 h/year	$289	
	Labour on-costs	20.5% × total labour	$2305	
	Total			$13 551
Overheads		27.5% × total labour activity		$3093
Travel costs	Meetings	5196 KM travelled/annual	$3741	
	Student support	600 KM travelled/annual	$432	
	Accommodation and meals		$1301	
	Total			$5474
Scholarship costs		$2,150 per person × 44 students		$94 600
Conference costs	Once every two years	$20 000 every two years		$10 000
Total ($AUD)		For cohort of 44 students		$126 718
Cost per student ($AUD)				$2880

(2) General practices—expected costs per one 18-month candidature			Item Cost ($AUD)	Total ($AUD)
(a) During the candidature^b^				
Student support	Fee assistance		$500	
Student training	Student training	MPA student training time^c^	$5216	
		Reception backfill: practice manager	$7565	
	Labour on-costs	20.5% × total labour	$2620	
	Total			$15 901
Overheads^d^				$3515
Study leave for students		7 days of study leave for student	$1387	
		Backfill cost during study leave^f^	$1883	
	Total			$3271
Total cost per student				$22 687
(b) Post candidature assuming wage increase and half day release for MPA duties				
Wage increase	MPA	Additional wages per year	$7168	
	Receptionist	Additional wages for ½ day receptionist	$31 156	
	Total			$38 324
Overheads				$8 746
Total recurrent yearly cost per graduate				$47 070
Cost per student 12 months post-graduation ($AUD)				$69 756

(3) MPA graduates—expected costs per one 18-month candidature			Economic costs^e^	
			Item cost ($AUD)	Total ($AUD)
Out of pocket costs				$850
Study and personal leave	Personal leave for MPA	Average 5 days per student: cost of forgone activities = 35% of wages	$297	
	Unpaid time for MPA	Average Total 330 h: cost of forgone activities = 35% of wages	$2582	
	Total			$2879
Cost per student ($AUD)				$3729

^a^Includes scholarships and administration for first year of commencing cohort (*n* = 44)

^b^Economic costs are opportunity costs/values of all resources by the Practice expended for MPA qualification.

^c^Practice Nurse conducts training during usual service, hence Practice Nurse is not diverted to training and incurs no opportunity cost. Any extra time for training considered negligible.

^d^Backfill economic costs calculated with respect to cash outlays for casual receptionists and the cost of directing permanent staff to backfill for receptionist. Calculated on MPA survey data. Backfill cash cost, the expected cash outlay for backfilling MPA student, accounting for the fact that some practices do not hire casual backfill.

^e^Economic costs are opportunity costs/values of all resources by the Practice expended for MPA qualification.

^f^Backfill economic costs calculated with respect to cash outlays for casual receptionists and the cost of directing permanent staff to backfill for receptionist. Calculated on MPA survey data. Backfill cash cost, the expected cash outlay for backfilling MPA student, accounting for the fact that some practices do not hire casual backfill

#### Potential revenues

Analysis also showed potential revenue increases for General Practices and students/graduates. However, there were no monetisable consequences for the PHN investment into the MPA Program given the PHN was never intended to be a monetary beneficiary of the MPA program. (Table 4 (1)).

**Table 4:** Monetisable consequences for General Practices and Medical Practice Assistants, post-graduation

Monetary consequences	
**(1) Hunter New England and Central Coast Primary Health Network**	No monetizable consequences
(2) GENERAL PRACTICES	

(a) Potential gross revenue from seeing extra patients per week (MBS 23 general consult = $38.75)			
	Extra patients per week (5 days)	Extra patients per year (52 weeks)	Potential annual gross revenue
Lowest	0	0	**$0**
Mid-point	6	312	**$12 090**
Highest	12	624	**$24 180**

(b1) Potential increase in gross revenue if practice nurse uses the half day of relief from lower order tasks to conduct health assessments and one form of care planning per health assessment (MBS item (703 + 721) OR (703 + 723))				
	MBS Item number	Revenue per assessment and plan	Reasonable number of tests performed per day/year [5 days × 52 weeks]	Potential annual gross revenue
Health assessment + GP management plan	703 + 721	$143.50 + $150.10 = **$293.60**	2 p/day × 5 days p/wk × 52 weeks = 520	**$152 672**
Health assessment + team care arrangement	703 + 723	$143.50 + $118.95 = **$262.45**	2 p/day × 5 days p/wk × 52 weeks = 520	**$136 474**
Total additional gross revenue per year			**$136 474–$152 672 (midpoint = $144 573)**	

(b2) Potential increases in gross revenue if instead of relieving the Practice Nurse, the MPA uses a portion of their 4 h of non-reception time to undertake additional billable clinical tests				
Test	MBS item number^b^	Revenue per test	Reasonable number of tests performed per day/ year	Gross revenue per year
Spirometry	11505	$42.80	2 p/day × 5 days p/wk × 52 weeks = 520	$22 256
Audiometry	11306	$22.80	6 p/day × 5 days p/wk × 52 weeks = 1560	$35 568
Pregnancy test	73806	$10.15	12 p/day × 5 days p/wk × 52 weeks = 3120	$31 668
Electrocardiography	11707	$19.15	6 p/day × 5 days p/wk × 52 weeks = 1560	$29 874
Ankle brachial index	11610	$66.30	8 p/day × 5 days p/wk × 52 weeks = 2080	$137 904
Visual Acuity	12326	$52.00	4 p/day × 5 days p/wk × 52 weeks = 1040	$54 080
Total additional gross revenue per year				**$22 332–$137 904**

(c) Potential net budget savings if the MPA backfilled the practice nurse (for half a day) whilst on annual and personal leave			
		Costs ($AUD)	Savings ($AUD)
Over 20 days annual leave			
Casual practice nurse cost	Annual Cost of hiring a casual PN for a full day for 100% of annual leave (20 days), plus overheads	$10 897	**$2332**
50% MPA and 50% casual receptionist	Cost of hiring casual receptionist to cover 50% of MPA day and a casual PN to cover the remaining 50% of the PN’s day, plus overheads	$8565	
Net saving	For 20 days per year (annual leave only)	[$10 897–$8565]	
Net savings per day	For 20 days per year (annual and personal leave)		**$117**
Over 35 days annual leave (20 days annual leave plus 15 days sick leave)			
Casual practice nurse cost	Annual Cost of hiring a casual Practice Nurse for 100% of annual leave (20 days) and 100% of personal leave (15 days) (plus overheads)	$19 069	**$4080**
50% MPA and 50% casual receptionist	Cost of hiring casual receptionist to cover 50% of MPA day and a casual PN to cover the remaining 50% of the PN’s day, plus overheads	$14 989	
Net saving	For 35 days per year (annual and personal leave)	[$19 069–$14 989]	
Net savings per day	For 35 days per year (annual and personal leave)		**$117**
(3) GRADUATES			
There is currently no set award for MPAs within general practice. The survey revealed a range between no increase in pay to a $3 increase in pay. The following calculations are based on the mid-point in the range and the aspirational rate which is halfway between a receptionist and an enrolled Practice Nurse wage			

		Per hour increase ($AUD)	Per week increase ($AUD)^a^	Per year increase ($AUD)
Wage increase	Aspirational	$3.00	$114	$5948
	Mid-point	$1.50	$57	$2974

^a^Annual wages have been calculated as 52.17857 weeks as prescribed by the Public Health System Nurses and Midwives Award 2021 (page 9) https://www.health.nsw.gov.au/careers/conditions/awards/nurses.pdf

^b^http://www.mbsonline.gov.au/internet/mbsonline/publishing.nsf/Content/Home

For general practices with graduated MPAs, potential extra revenues were recognised where the graduate could: (a) conduct extra revenue earning tasks as qualified by the course; or (b) replace practice nurses on lower-level tasks, freeing the nurses for higher revenue services such as creating GP management plans. Scenario analysis found potential annual gross extra revenue of $136 474 to $152 672 or a mid-point of $144 673 per practice (Table 5, Modelled Scenario A). On this basis, general practices could recoup their MPA investments within one year. Potential increases in remuneration exist for MPA graduates. If able to negotiate a gross pay increase of between $1.50 and $3.00 per hour, MPA graduates will have a gross income increase of between $2974 and $5948 per annum. Given their MPA related economic costs are $3729, prospective students can expect to recoup their full investment within 2 years of graduation. Economic costs and potential extra revenues are described in detail in the Appendices.

**Table 5:** Cost and consequences for the PHN, General Practices and MPA graduates

Perspective	Costs	Value	Potential monetisable consequences	**Mid-point value** *(sensitivity analysis: min–max values*)
PHN	Cost of MPA Program Coordination and scholarship subsidy (2018 cohort of 44 students)	**$126 718**	No monetisable consequences	
General Practices	Initial course and training investment	**$22 687**	Modelled scenario A (a) Six extra patients per week (0 to 12 patients)	**$12 090** *($0–$24 180)*
	Optimal utilisation costs per annum per MPA graduate	**$47 070**	(b) Expected additional Practice Nurse- generated gross revenue from health assessments ($0 to maximum)	**$144 573** *($0–$152 627)*
	Total cost at 1-year post-graduation	**$69 756**	(c) Total gross revenue 1-year post-graduation (a + b)	**$156 663** *($0–$176 807)*
			(d) Total net revenue 1-year post-graduation (c -$69 756)	**$86 907** *(****−****$69 756–$107 051)*
			Modelled scenario B (a) Six extra patients per week (b) Additional MPA generated gross revenue from extra billable tests (c) Total gross revenue 1-year post-graduation (a + b) d) Total net revenue 1-year post-graduation (c – $69 756)	**$12 090** *($0–$24 180)* **$68 952** *($0–$137,904)* **$81 042** *($0–$162 084)* **$11 286** *(-$69 756–$92 328)*
MPA graduates	Total course-related costs	$3729	Modelled scenario C (a) Annual increase in wages per year post graduation	**$2974 *(****$0–$5948)*
	Possible offsets: Aspirational annual wage increase	**$5948**	(b) Total net MPA outcome 1-year post-graduation (a–$3729)	**$-755** *(-$3729–$2219)*
	Midpoint Lowest annual wage increase	**$2974 -** **$0**	(c) Total net MPA outcome 2 years post-graduation ((2 × a) -$3729)	**$2219** *( -$3729–$8167)*

^a^Intervals reflect the range of monetary income/cost between not supporting an MPA and supporting an MPA

### Narrative

Table 6 presents the narrative of the MPA Program which summarises the pathway to impact from *need* for the MPA program through to the *impacts* on general practices and MPA graduates, as depicted in the Program Logic Model (Fig. 1). The narrative provides the context against which the results from the Payback and cost-consequence analysis can be interpreted.

**Table 6:** Narrative of the MPA Program

Need Workforce shortages in rural and remote health care delivery pose a persistent issue across Australia and can result in suboptimal and high-cost care, particularly in respect to primary practice. Skill shortages, retention issues, smaller staff numbers and a broad range of non-specialised tasks being undertaken by specialist roles, can lead to longer wait times and shorter consult times for patients, and increased costs to the practice. Various initiatives have been implemented to increase numbers and retention of health care providers and improve efficiencies in health care delivery. Optimising GP and PN time and supporting them to work at the top of their scope are critical to increasing efficiencies, improving cost effectiveness and enhancing patient care. Additionally, evaluation (including economic and impact assessment) is critical to optimizing the outcomes and impacts of such initiatives and inform future programs accordingly
Program response To address these needs, the Hunter New England and Central Coast Primary Health Network (HNECC PHN) implemented an initiative to upskill general practice administrative staff (mainly receptionists or practice managers) in a range of appropriate clinical and high-end administrative tasks. The aims of the initiative included: broadening the scope of practice managers and practice administration staff, building health workforce capability, reducing the impact of skill shortages in primary practice, boosting productivity within practices through more cost-effective delivery of clinical services, increasing job and role satisfaction for practice staff and ensuring roles are working at the top of their skill level and qualifications. The HNECC PHN entered into an arrangement with UNE Partnerships—an entity certified to deliver the MPA Course; engaged practices to encourage their administrative staff to undertake an MPA Certificate (Cert IV) and provide the required on-the-job supervision; subsidised course fees for administrative staff from local general practices and Aboriginal medical services to undertake the MPA Cert IV, and coordinated and resourced regular MPA Conferences to facilitate learnings from the initiative, created a network of MPA practitioners in the region and have contributed to efforts to have the MPA role officially recognised and a recognised national award created for medical practice assistants
Program outcomes Whilst the program initiatives and the full impact of the MPA program is yet to be fully realised, a multitude of desired outcomes have been achieved. Forty four students have graduated from the MPA course (as of 1 June 2021) as more highly skilled practice staff, 62 general practices have been recruited into the program initiative in 2018 and 2019, 62 scholarships have been granted to general practice staff in the region, MPA program expression of interest processes have been established, along with a growing network of graduates. Additional support packages for Aboriginal MPA students and a series of new resources and further development opportunities have been provided (i.e., webinars, conferences etc.), including pathways to accrue continuing professional development (CPD) points. A University of New England (UNE) booklet has been produced and progress towards establishing new practice roles and award rates for MPA qualified administrators is underway
Impact The program initiative has been successful in generating a range of economic, knowledge, capacity building, practice, staff and community and patient impacts, as evidenced in the Payback metrics (Table 3). Most significantly, the program has resulted in greater work satisfaction for practice staff, and improved ability of practices to provide better patient care. GPs and PNs reported greater work satisfaction, GPs reported an increase in resources to provide patient care and PNs reported less stress and reduced workloads*.* MPA graduates felt the course had benefitted them professionally and personally with 80% reporting higher job satisfaction and confidence and being able to fill in for a PN when required. Other benefits included increased knowledge, increased hours and pay, more variety and capacity in work tasks, improved relationships with colleagues and patients as exemplified by one graduate “*my relationships with other staff have been better as I understand everything a lot more and I can help everyone out*” Graduates also reported the ability to work at a higher level (“*I get to do more things now*”) get more hours of work compared to previously, and “*enjoying the challenge and finding it more satisfying*” Additionally, MPA graduates felt they had personally gained new perspectives and that this knowledge was also beneficial in their personal lives e.g., enabling assistance with family members’ health and having “*a lot more confidence with my grandkids if they are unwell”* Critically, undertaking the MPA has sufficiently built their confidence to undertake further tertiary studies- primarily in health care: “*Since completing the course it has given me confidence to go further with my studies”*. Forty five percent have gone on to study nursing, social work, practice management and paramedicine. This is important for reducing skill shortages in the region and creating progressive career pathways that further add to job satisfaction, ultimately aiding in retention of skilled staff in rural and regional areas Positive impacts of the program were also seen within practices, “*Having a MPA has been a valuable asset throughout our entire practice*” as demonstrated through the cost and time efficiencies gained by practices not requiring additional temporary fill in staff when their existing staff are unwell or on leave. MPA graduates were able to provide sufficient coverage: “*On days when we are short of nurses our MPA is able to contribute to our workload with being able to triage, complete sterilization and assist GPs with procedures*” Practices also benefitted through more effective use of PN and GP time, value adding and improving productivity “*our MPA has been very beneficial to our clinical area leaving more time for registered nurses to deal with more urgent situations on the day*”. They have also allowed streamlining of tasks and responsibilities: *“…we have our MPA assist with diabetes clinics”, reducing key bottlenecks and through associated flow-on positive impacts on other practice staff and patients* *“By helping out with the extra duties, it has taken the pressure off the nursing staff”* *“They are a really great resource to reduce workload and stress of nursing staff* -*this helps our treatment room RN with the waiting time for patients”* Improving practice staff capacity and knowledge and reducing staff stress has additional flow on benefits for patients and the broader community. For this study patient surveys were not undertaken, however feedback was provided by practice staff regarding their perspectives on improved patient care. Survey and interviews revealed that patients of practices with an MPA graduate, experienced reduced waiting times, improved care, and seamless continuity of care—“*we are able to offer a better health outcome and continuation of care as a result of improved patient rapport and more meaningful engagement between staff, leading to better health outcomes for patients.”* Another reported “*patients have had one on one time with me and have commented on how lovely it is to see me out in the nurses’ station*” while another said “*having a broader knowledge base and being able to relate that to the patient and making them more at ease. It extends their contact time with someone while they are in the clinic”.* Shorter wait times and less seemingly rushed consultations, result in greater patient satisfaction, improved triage and patient care, and reduce risks of cross patient infection Overall, despite the short lag time between MPA graduation and this survey (only 22 graduates back working in the practice post-graduation) the HNECC PHN initiative has had significant impact for the limited number of graduates and practices included in the assessment. Whilst the program is new and still being refined, demonstrable impact has already been realised and captured through this assessment The most significant impact of the MPA program in this cohort is the increase in job satisfaction of broader practice staff- including practice managers, GPs and PNs in addition to MPA graduates. Job satisfaction is a key pillar in rural and remote workforce retention and the flow on effects of introducing tertiary study to the existing workforce and encouraging further upskilling and study has promising implications for further growing and retaining the health workforce in the Hunter, New England and Central Coast regions of NSW but also holds promise for other rural and regional locations in Australia

### Suitability of FAIT

With regards to the novel application of FAIT to a health services led program, the FAIT method was able to be applied to the MPA Program without any specific customisation over and above what would be expected from its application to a research program. Although the PLM was applied retrospectively, it proved to be useful for: (i) documenting the pathway between the MPA Program and its impact on practices, their staff, MPA students and graduates; (ii) identifying metrics that could evidence impact from the MPA Program; and (iii) raising awareness within the PHN team as to other benefits of the MPA Program that had not been previously considered such as a reduction in stress for PNs or certain measures of increased productivity such as additional patients seen in the practice per day. The economic analysis gave transparency to the economic cost involved in the delivery of the MPA Program from the perspective of the PHN, participating practices and MPA students. It was also able to project the monetary value of some of the potential consequences of the investment. The narrative articulated the pathway from the need for the MPA Program (a solution to workforce issues within rural general practice) to the impact; and expressed benefits of the MPA Program that could not be expressed in quantitative terms such as “improved relationships between MPA graduates and their colleagues and patients” and “confidence to undertake further tertiary studies- primarily in health care”.

## Discussion

### Impact of MPA program

The impact assessment found the MPA Program to be a feasible, economically viable strategy for rural general practices in Australia to address workforce shortages and retention issues. When appropriately managed, the program increased potential for revenue generation and improved patient care, and increased job satisfaction for MPA graduates, PNs and GPs, an outcome linked to improved workforce retention [1, 9, 29]. The MPA Program provided participating practices with a multi-skilled, flexible resource who could be used in an ad-hoc manner to fill in for PNs, where appropriate, or in a more systematic way by backfilling PNs on leave, undertaking scheduled clinical tasks that attract additional revenue for the practice, or providing more hands-on care to patients. MPA graduates were also able to free up PNs from non-revenue generating tasks to undertake more complex patient-oriented activities like health assessments and care plans, which provide more comprehensive care to patients in at risk groups and with complex needs, while generating revenue for the practice. If utilised to capacity, a practice, and its MPA graduates whose additional training was appropriately recognised with a salary increase, could recoup their respective investments in 12–24 months and practices could go on to increase their profits and clinic throughputs over subsequent years.

Qualitative findings suggested other benefits including reduced workload, reduced stress, and reduced pressure on PNs in the practice. The MPA graduates also benefited through their enhanced market value (through their upskilling and increased confidence) and increased job satisfaction associated with undertaking more meaningful tasks. In addition to existing strategies like recruiting more overseas trained doctors, and training more local doctors [13, 30], the MPA Program value adds to such initiatives through utilising an existing, non-scarce medical administration workforce to allows PNs and GPs to work at the top of their scope, relieve workload pressure during peak periods and provides education pathways for administration staff, all key factors in improving regional and remote workforce retention and maximising cost efficiency and workforce capacity [13, 16, 17]. The MPA Program would be transferable to other countries with a fee-for-service arrangement in primary care and who face the same workforce issues that prohibit adequate servicing of rural and regional populations. It is particularly useful for busy practices, those with labour shortages, and large practices where there are greater economies of scale to be gained by having an MPA who can be utilised to their maximum capacity.

### Other insights about the MPA program

The impact assessment also revealed a large variation in the utilisation and remuneration of MPAs, resulting in a variance in the anticipated benefits for participating practices. This suggests that greater consistency in the utilisation and remuneration of MPA graduates and more supported collaborative integration planning involving all practice staff and the PHN, could optimise the benefits of the program. In addition to being adequately renumerated for their additional skills, MPA graduates also suggested that greater recognition of the role, a dedicated award, and greater direction on how to manage this role within the practice team would improve the overall MPA experience. Support from the PHN through providing relevant good practice models and exempler case studies of succesful MPA graduate reintegration and utilisation would be of benefit. Additionally, the impact assessment highlighted the importance of embedding impact planning and evaluation processes upfront to minimise data collection burden and costs and optimise impact. Additional file 5 summarises recommendations for optimising the benefits and impacts from the MPA Program.

### Strength of the study

The comprehensive collection of evidence and its analysis, as per the FAIT methods, was a key strength of the study. The co-development of the PLM allowed the project team to fully consider all components and perspectives of the MPA Program and develop appropriate metrics to measure its impact. The Payback metrics enabled a comprehensive assessment of the full range of possible impacts of the MPA Program. The CCA gave transparency to the investment from all three perspectives (PHN, general practices and MPA students) informing future implementation and scale-up of the initiative. The narrative brought to light the benefits of the MPA course from the perspective of the MPA graduates and their voices were given prominence.

The collaborative research approach provided capacity and capability benefits to PHN staff. PHN staff improved their confidence and skills in measuring and reporting impact in a multi-dimensional way. PHN staff also co-produced the research and have benefitted from the networking and learning opportunities with HMRI staff.

### Limitations and implications

COVID-19 is likely to have impacted on interview response rates, thereby limiting the representativeness of the results. New mandated infection control measures and increased demand for consultations meant that general practices and AMSs were under additional pressure between November 2020 and February 2021, the period when the survey was open. While remedial measures were taken to increase response rates (e.g., extending survey deadline and offering incentives), they remained below an ideal level. It is also likely that practices who responded to the survey represented practices that had a more positive MPA experience than non-responding practices. This could have biased the results in favour of greater positive impact from the MPA program.

COVID-19 also impacted MPA students in the 2019 and 2020 cohorts increasing their workload and their course time, thus reducing the number of participating practices with returning MPA graduates. COVID-19 also limited MPA graduates’ abilities to fully utilise their skills given the high volume of phone consults during this period. The timing of this impact assessment did not provide sufficient lag to realise longer term benefits of the MPA Program such as reduction in workforce shortages.

Potential monetisable consequences used in the modelling were not exhaustive. According to survey results and expert input, MPAs can also increase gross revenue through paid quality improvement activities (e.g. undertaking Practice Incentive Program (PIP) quality improvement measures including height and weight measurements for all patients) which attract a government payment [27] that many practices currently forgo due to a lack of nurses’ time to complete such activities. These other potential consequences required a large number of assumptions to support the modelling, and consequently were not included.

The absence of Aboriginal and/or Torres Strait Islander MPA students, MPA graduates, or staff from Aboriginal Medical Services gives no visibility to the impact of the MPA Program on their operations and means that their perspectives are missing from this study. This limits the applicability of these findings to Australia’s First Nation peoples and the services controlled by their communities. Low numbers of Aboriginal MPA students in the course is thought to be partially due to a preference for attaining the Aboriginal Health Worker qualification rather than the MPA qualification and is an area worthy of further review and discussion. The absence of direct feedback from patients, consumers and caregivers is a further limitation that was unable to be addressed within the resources and timing of this study. Future evaluations of the MPA Program should consider the inclusion of patient perspectives and views to provide a more holistic evaluation.

## Conclusions

The HNECC PHN initiative and resultant outcomes and impacts associated with regional general practice administrative staff undertaking the MPA Program have been significantly beneficial to a number of participating practices Critically, the impact assessement of the MPA program indicates there is room for improving utilisation of MPA graduates in the workplace and that the program has broader long term potential to mitigate the impacts of regional workforce shortages and low retention, improve primary practice efficiences and gross revenue and further grow and upskill the regional health workforce.

## Supplementary Information

**Additional file 1**: The Framework to Assess the Impact of Translational Health Research. Description: A detailed description of the FAIT method.

**Additional file 2**: Unit Costs by Perspective. Description: Detailed unit costing of the MPA program resources from HNECC PHN, participating General Practices and MPA students.

**Additional file 3**: Supplementary Methods: Assumptions associated with estimating program costs and detailed survey and interview data. Description: Overview of assumptions and methods for establishing program costs and a detailed description of the survey and interview process.

**Additional file 4**: Consequence and Revenue Modelling. Description: Detailed description of the methods for consequence modelling and revenue scenarios for general practices with a returning MPA graduate.

**Additional file 5**: Recommendations for optimising the benefits and impacts from the HNECC PHN Medical Practise Assisting Program. Description: Outline of key recommendations for optimising the benefits and impacts from the HNECC PHN Medical Practise Assisting Program as demonstrated through the FAIT Impact assessment.

## Data Availability

The datasets used and/or analysed during the current study are available from the corresponding author on reasonable request.

## Abbreviations

Ahpra: Australian Health Practitioners Regulation Authority
AMS: Aboriginal Medical Services
AUD: Australian Dollar
CQI: Continuous Quality Improvement
EOI: Expression of interest
FAIT: Framework to Assess the Impact of Translational Health Research
GP: General practitioner
HMR: Health and Medical Research
HMRI: Hunter Medical Research Institute
HNECC PHN: Hunter New England and Central Coast Primary Health Network
MBS: Medicare Benefits Scheme
MPA: Medical Practice Assisting
MPA Program: Certificate IV in Medical Practice Assisting
PDSA: Plan Do Study and Act
PHN: Primary Health Network
PLM: Program Logic Model
PM: Practice Manager
PN: Practice Nurse
Practice: General practice
R&R: Rural and regional
REDCap: Research Electronic Data Capture Survey Platform
UNE: University of New England

## Footnotes

1 Monetisable consequences refer to consequences that can be converted into a monetary/dollar value. For example, a consequence of graduation from the MPA Program is an increased hourly wage which can be monetised.
