# Supplementary material for: Impact assessment of the medical practice assisting (MPA) program in general practice in the hunter New England and central coast regions of Australia
Source: Hum Resour Health. 2022 Dec 5;20:81. doi: 10.1186/s12960-022-00781-6 (PMC9721062; doi:10.1186/s12960-022-00781-6)
Supplement: Supplementary file 1 — Additional file 1: The Framework to Assess the Impact of Translational Health Research. Description: A detailed description of the FAIT method. [file 12960_2022_781_MOESM1_ESM.docx]

**Additional File 1: The Framework to Assess the Impact of Translational Health Research**

The Framework to Assess the Impact of Translational Health Research (FAIT) is a framework that was originally designed to both measure and encourage research translation and research impact. In this study it is being applied to an interventional program, rather than a research project. The original design and implementation of FAIT can be found at

The FAIT model was developed by the HMRI team by reviewing, combining and modifying three existing and validated techniques for research impact assessment; Payback, economic analysis and narratives (see Figure 1 for a brief description of what each method achieves). These methods can also be used to understand the impact of an intervention, looking to understand its impact within domains of benefit, the return on the implementation costs and an understanding of the broader impact on key end users who stand to benefit from the intervention.

Figure 1 Framework to Assess the Impact of Translational health research (FAIT) model


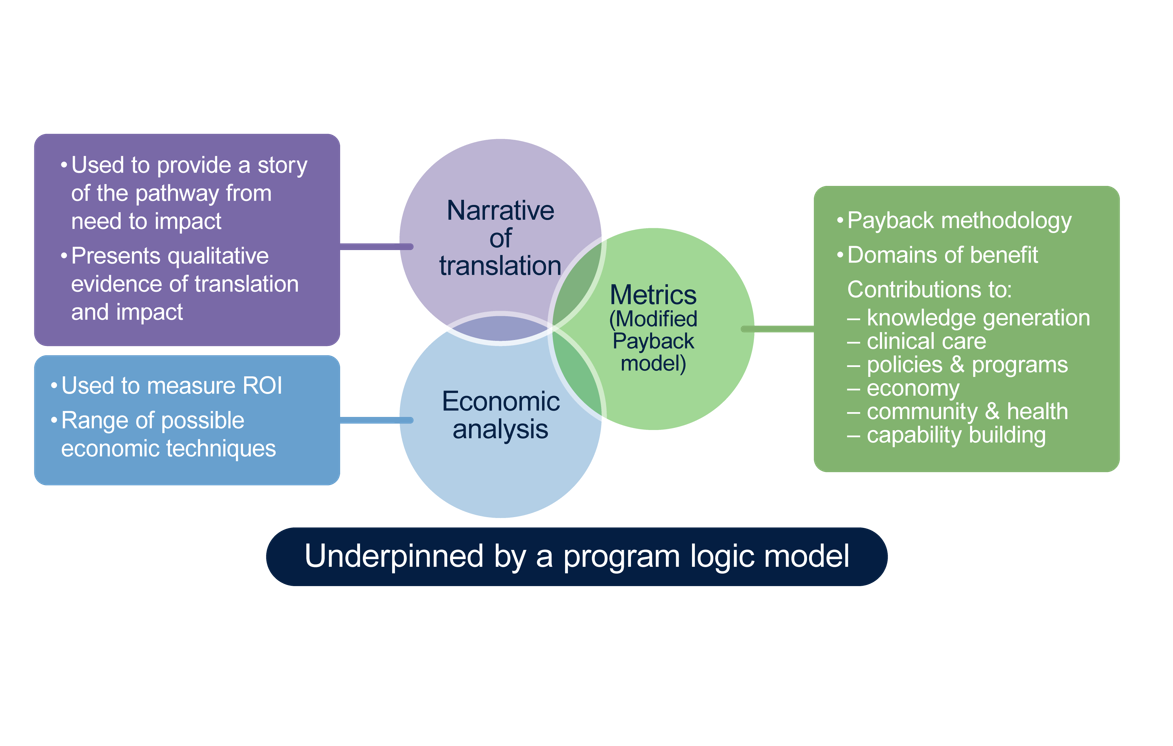


**Development of a Program Logic Model**

The first step in the implementation of FAIT is the construction of a modified program logic model (PLM) which serves as the foundation on which to build the complete impact assessment. Logic models are a simple, transparent, and systematic way for researchers or practitioners to articulate their understanding of the relationships between the need or problem they are trying to address, their planned activities and the impact they hope to realise.

In the context of FAIT, the PLM should be thought of as a strategic map that links each individual stage of the research/ program intervention and impact assessment into a cohesive whole. The logic model provides a road map for the development of impact metrics, the collection of evidence for these metrics, the activities and impacts to monetise for the economic evaluation and the pathway from need to impact, the basis of the narrative.

FAIT’s modified program logic model outlines six core elements:

1. The **need** being addressed by the research or program intervention.
2. The specific **aims** of the research or program intervention.
3. The research/program **activities** that will be undertaken to meet these aims.
4. The expected **outputs** from those activities
5. The **end-users** who will implement and/or benefit from these outputs.
6. The anticipated **impact** of the research or program intervention.

**The Payback Framework**

***Selecting Domains of Benefit***

Payback uses different “domains of benefit” to highlight the various areas where a particular research project or intervention program could have impact. The original Payback had five domains of benefit (Knowledge Advancement, Clinical Implementation, Community Benefit, Legislation and Policy, and Economic Impact) which were designed to cover a wide range of perspectives. Domains can also be added and customised to the project or program. Conceptually, Payback was modified for application within FAIT. It has been modified in three ways:

1. to be applied to a prospective rather than a retrospective measurement framework.
2. to populate the domains of benefit with quantitative metrics, rather than qualitative interview data.
3. to include other relevant domains of benefit such as capacity building and health system improvement that may be relevant to the research being assessed.

While impact does not necessarily need to be categorized in terms of these domains, doing so allows for a more straightforward process to create metrics. Producing evidence across multiple categories helps ensure that the impact of the research or implementation program can be readily understood by end-users across many different sectors. The use of the Payback scorecard allows various groups to effectively evaluate the overall impact of the research in ways that are most relevant to them.

***Developing Impact Metrics***

The next step in FAIT is the selection of impact metrics and creation of a scorecard. Careful consideration must be given to ensure that there is a clear rationale for why a certain metric merits measurement and how it will generate meaningful evidence about the impact of the research or implementation program. It is also important to ask which stakeholders may be interested in each impact selected. Keeping these stakeholders in mind, it is important to understand whether the impact can be effectively assessed within the domains that matter to those stakeholders. The categorization process is intended to help clarify the process of developing metrics that will maximize the demonstrable impact of the research or implementation program. There are standrad metrics for research projects, less so for implementation programs so many of the metrics will need to be customised. Here is one list that could be useful:

- [Mapping outcomes for social investment](https://www.thinknpc.org/resource-hub/mapping-outcomes-for-social-investment/) (A selection of examples for creating outcomes measures based on different project types)

**Economic Analysis**

Economic evaluation is an essential part of the implementation of FAIT, but it is also undoubtedly one of the most complicated steps in the process. In concert with the development of impact metrics, stakeholder engagement is a central reason for conducting an economic evaluation. Those who plan, provide, receive, are affected by, or pay for research want to be able to measure the merits of that research, and a measurement of merit that is understood by almost all stakeholder groups is monetary value. Economic evaluation can take a wide variety of forms, but the goal is always the same: to estimate a value for a given intervention be it service or product after taking into account all of the inputs and outputs associated with it, so that it can be compared with other possible alternative courses of action. This allows decision-makers and other stakeholders to more easily identify which alternatives are worth their attention and funding, and which are not likely to be productive investments.

Of the different varieties of economic evaluation, Cost-Benefit Analysis (CBA) is one of the most commonly used, and it is also the foundation for the economic evaluation aspect of FAIT. While other evaluation techniques compare costs in monetary terms with benefits that have specific non-monetary measurements (eg. number of patients screened), CBA attempts to translate these benefits into monetary terms as well, in order to present results in the most straightforward possible way. Since FAIT is designed to present research and program impact in a way that is accessible to all stakeholders, translating benefits into monetary terms is helpful because it allows for an assessment of impacts across different domains using a single measurement scale, which makes the results more broadly applicable. Despite the position of CBA as the “gold standard” method for economic evaluation within FAIT, there are some research projects or intervention programs that may require a different evaluation technique - simply because a CBA is out of reach due to a lack of data or inappropriate in terms of timing as the downstream benefits/consequences that can be monetised have not yet occured. For research/programs like this, a Cost-Consequence Analysis (CCA) can replace the CBA to provide a meaningful economic evaluation, although it will also necessarily result in a slightly modified application of FAIT.

A CCA will still involve converting benefits into monetary values wherever possible, but it will also include the ones for which this is not feasibly possible, using their original measurements or values without attempting to aggregate across dimensions. This format is designed to incorporate benefits such as humanistic outcomes (eg.work satisfaction; social function) and benefits that are not expected to be fully realized until long into the future (eg.patient health outcomes). In essence, the CCA is intended to present a transparent account of costs and benefits across many different dimensions using their original measurements, allowing decision-makers to see clearly what types of information are included and omitted, and where information is quantitative or qualitative. Within FAIT, the Payback table records the all consequences using quantified metrics, leaving the monetisable impacts to be included in the cost-consequence analysis.

***The Determination of Research and Implementation Costs***

The actual determination of the costs and benefits of the research/implementation program is the step that will determine the eventual quality of the economic analysis. To establish what costs are “extra” as a result of the research or implementation project, it is also important to establish a counterfactual, by determining the existing cost structures and how the research or implementation program would alter them. Another important concept to consider during cost determination is “opportunity costs”. An opportunity cost is any loss of a potential gain when one alternative is chosen over another. In health research, this can take many forms: extra labour costs of backfilling the MPA student could have gone instead to other forms of care.

Once a primary set of likely costs from the different perspectives has been determined, the next step is to determine their actual monetary values. This step requires the evaluator to obtain the best possible estimations, but there are often helpful resources that contain the necessary information, especially when it comes to research in healthcare settings or implementation programs. It is not anticipated that a complete and perfectly accurate set of costs will be a realistic goal for most research studies or implementation programs but rather a reasonably strong estimation.

In contrast, many benefits derived from health research or program inmplementation are not as easily translated into monetary value without making a range of assumptions. Unlike with costs, the benefits of a given project or program can take a very wide variety of forms, and each type of benefit will have different applicable strategies for valuation. The creation of microcosting models, where every individual cost and benefit factor is measured in detail, is possible, but is usually resource intensive and requires significant primary data collection. In addition to the valuation methods introduced above, there are several important technical steps to consider that will increase the accuracy of the economic evaluation.

***Projection/Simulation/Time Horizon***

Projections of how the costs and benefits of a given project/program will occur and change over time are critical to establish realistic valuations for impact. Time horizons should be chosen so that they are long enough to capture most of the meaningful impacts, but not so long as to rely upon unrealistic assumptions for what the situation will be into the future. To simulate time, costs and benefits can be estimated in cycles, meaning that for chosen blocks of time after implementation (1 month, 6 months, 1 year - depending on the nature of the project), we measure a value for costs and benefits based on available projections. This means that for every cycle measured we will have a calculated value to add to the economic evaluation.

***Nominal dollars***

In initial monetary measurements, most values are reported in what are called “nominal dollars”. Nominal dollars simply indicate the value in terms of money at the time that the measurement was taken. However, some measurements may have been taken in different years, and due to inflation, nominal values from one year to the next cannot be accurately compared. To correct for this, all monetary values should be indexed to “real dollars” based on a chosen year (usually the current year) prior to reporting. Real dollars is the value of a cost or benefit after it has been properly adjusted for inflation, allowing all monetary values to be compared as if they accrued at the same time.

***Sensitivity analysis***

Regardless of how values are obtained, they will always have an inherent margin of error. This is especially true in health research or program evaluation, where many different factors can contribute to the valuation of a given cost or benefit. To account for this margin of error, a sensitivity analysis should be performed as the last step in any economic evaluation. Sensitivity analyses attempt to measure how susceptible a presented economic analysis might be to fluctuations in methodological choices (including which discount rates were used) or valuation decisions by including measures of uncertainty into the economic evaluation itself. The purpose of a sensitivity analysis is not to determine exactly how inaccurate the values are, but rather to present stakeholders with an idea of how far the final results presented from an economic evaluation could deviate from the true societal value.

The first step in performing a sensitivity analysis is to determine a realistic range of possibilities for each value included in the economic analysis. Some values, such as those obtained from microcosting studies, will already be reported with an uncertainty range, but in other cases it is also acceptable to estimate a plausible range based on expertise and experience with the subject matter. Once these ranges of uncertainty are determined, values can be set at the low and high ends to see what kind of changes are caused in the economic evaluation.

Although a methodologically sound economic evaluation is a powerful tool for informing decision-makers, economic evaluations do have some known limitations. Economic modelling is frequently based upon simplifying assumptions, and some monetary values placed on benefits can be contentious. It is also extremely unlikely that any impact evaluation methodology will capture the entirety of a project’s impact across all of society. To mitigate the drawbacks of economic evaluation while still including the decision-making insights provided, the FAIT scorecard presents the economic alongside its other two core assessment components in order to deliver a well-rounded overview that both appeals to the widest possible range of stakeholders and meets their various informational needs.

**Narratives**

To complement the insights provided by the quantified metrics using the modified Payback framework and economic analysis; and to give some context to the pathway to impact, FAIT employs a third method of impact assessment in the form of narratives. Within FAIT, narratives fulfill an important role. They allow you to:

- tell the story of the research or implementation program
- describe how the impact was generated
- evidence impacts that cannot be quantified or monetised (impacts best expressed qualitatively)

The narrative method allows presentation of qualitative evidence of impacts such as quotes from end users. This type of evidence, where benefits are expressed from the perspective of the beneficiaries and in their words, can be quite powerful for funders and policymakers and others wanting to understand the research/implementation program. It helps to bring the human element to the impact.

Narratives are also very useful for telling the story about the research/implementation program and brings it to life.

When constructing a narrative the first step should be to consider the evidence presented by the other two methods. While the narrative should be supported by this evidence, they also provide an opportunity to describe the pathways or processes that contributed to the impact of the research or implementation project that the other two methods may not have covered. By writing a narrative, the goal is to build a qualitative framework around the other impact assessment findings, which allow them to be placed more coherently within the context of the research/implementation program and provides an opportunity to explain variances in research costs, outputs and impacts in more detail. Narratives also benefit from the addition of evidence of impact in the form of quotes or interview findings from the actual end users of your research.
