# Supplementary material for: Impact assessment of the medical practice assisting (MPA) program in general practice in the hunter New England and central coast regions of Australia
Source: Hum Resour Health. 2022 Dec 5;20:81. doi: 10.1186/s12960-022-00781-6 (PMC9721062; doi:10.1186/s12960-022-00781-6)
Supplement: Supplementary file 2 — Additional file 2: Unit Costs by Perspective. Description: Detailed unit costing of the MPA program resources from HNECC PHN, participating General Practices and MPA students. [file 12960_2022_781_MOESM2_ESM.docx]

**Additional file 2: Unit Costs by Perspective**

*Costing of the MPA program resources from HNECC PHN, participating General Practices and MPA students*

**
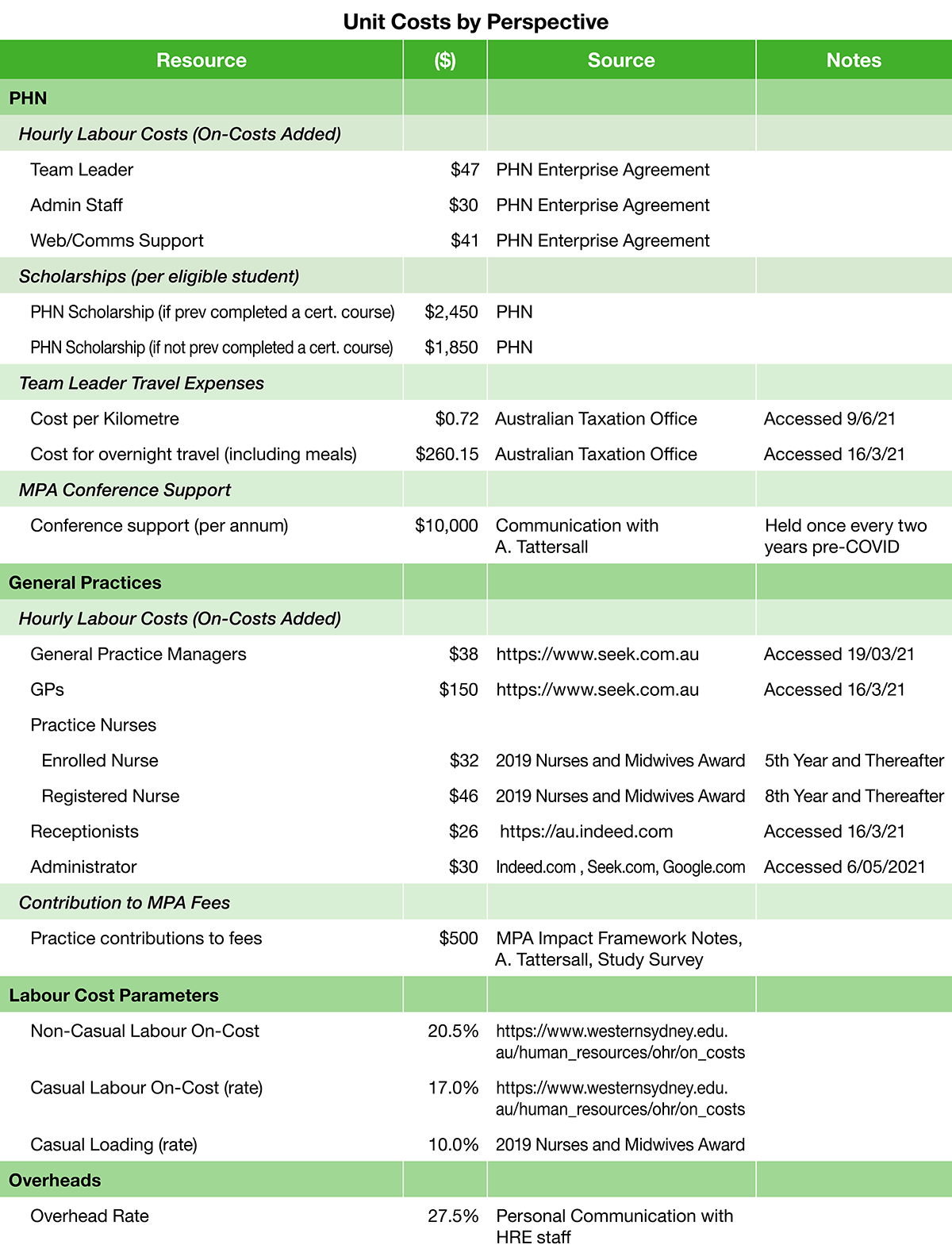
**
