# Supplementary material for: Impact assessment of the medical practice assisting (MPA) program in general practice in the hunter New England and central coast regions of Australia
Source: Hum Resour Health. 2022 Dec 5;20:81. doi: 10.1186/s12960-022-00781-6 (PMC9721062; doi:10.1186/s12960-022-00781-6)
Supplement: Supplementary file 3 — Additional file 3: Supplementary Methods: Assumptions associated with estimating program costs and detailed survey and interview data. Description: Overview of assumptions and methods for establishing program costs and a detailed description of the survey and interview process. [file 12960_2022_781_MOESM3_ESM.docx]

***Additional file 3 –*** ***Supplementary Methods (Costing and data collection)***

(A) Program Costing Methods

For PHN or general practice activities, the costs of overheads (electricity, water, security, building maintenance etc) and oncosts (superannuation, leave etc.) were added to labour costs at a rate of 27.5% for overheads and 20.5% for oncosts. Wage values were obtained from the PHN enterprise agreement, published awards or employment sites. Travel costs were based on allowances published by the Australian Taxation Office. PHN funded scholarships could be awarded at levels of $1,850 for persons who had previously completed a certificate course to $2,450 for persons who had not. As exact data was not available on the breakdown of scholarship payments, it was assumed that 50% of students received a full scholarship and 50% the lower value scholarship.

The value placed on student study time, either paid or unpaid, was set at 30% of students’ pay, as suggested by the Australian Safety and Compensation Council^[[1]](#footnote-1)^. The value for student study leave was estimated with an algorithm recognising costs of (a) hiring casual replacements or (b) allocating student duties to existing staff. Costs of PHN scholarships, general practice student support, and PHN support for the Bi-Annual MPA Conference were gathered from the PHN MPA Manager and triangulated with survey data. A Practice Nurse was assumed to be the supervisor of MPA students. The costing assumed the wage of a registered nurse after their 8^th^ year of practice (AUD $46 per hour).

Post-graduation, ongoing costs for practices for the MPA graduate included increased wages and the cost of additional receptionist wages required to relieve the MPA graduate, allowing them to utilise their new skills in the practice for 50% of their day, equivalent to 4 hours per day. A cost model was constructed in a Microsoft EXCEL workbook^[[2]](#footnote-2)^, linking unit costs with relevant gathered data to arrive at aggregated costs for the PHN and expected aggregated costs for general practices and students.

Costs for the PHN were collected pre-COVID and are reported as the ‘cost per graduate’ using the aggregate cost for the 2018 cohort of 44 starting students as the base. Aggregated costs for general practices and students were also expressed as ‘cost per graduate’. Costs to the University of New England for course administration and workshops production and the course subsidy cost to the NSW government were excluded from the study, as these perspectives were out of the scope of this evaluation. Costs were expressed in 2020-21 Australian dollars.

Survey and interview methods

Survey data collection went from 2 October 2020 to 14 February 2021. Practices were recruited via an invitation letter, ethics-approved Organisational Information Sheet, and a consent form. General Practices completed, signed, and returned the Organisational Consent Form with the name and contact details of the nominated contact person from their practice. This person was emailed a survey link to be distributed to eligible practice staff. Informed consent was obtained from all online participants. MPA Graduates were recruited directly by the MPA Coordinator . They completed and returned a Participant Information Sheet before they were contacted by a member of the HMRI team, and a telephone interview scheduled. Data was cleaned and analysed in SPSS (Version 19)^[[3]](#footnote-3)^. Given the small number of completed surveys, analysis was based on frequencies.

Additional practice and process information was gathered from the MPA coordinator and the HNECC PHN Primary Care Improvement Officer via emails and Zoom discussions. Both had intimate knowledge of the general practices involved in the MPA Program to supplement the data collected from survey participants. Zoom discussions were taped and transcribed. The suitability of the FAIT methodology was assessed through ongoing discussions and reflections of the multi-disciplinary project team (i.e., the authors). This group included PHN staff who were novel users of FAIT and HMRI staff who had experience applying the FAIT methodology in other contexts. The suitability of FAIT is also evidenced by the results that were generated through the application of FAIT to this project

1. [↑](#footnote-ref-1)
2. [↑](#footnote-ref-2)
3. [↑](#footnote-ref-3)
