# Supplementary material for: Impact assessment of the medical practice assisting (MPA) program in general practice in the hunter New England and central coast regions of Australia
Source: Hum Resour Health. 2022 Dec 5;20:81. doi: 10.1186/s12960-022-00781-6 (PMC9721062; doi:10.1186/s12960-022-00781-6)
Supplement: Supplementary file 4 — Additional file 4: Consequence and Revenue Modelling. Description: Detailed description of the methods for consequence modelling and revenue scenarios for general practices with a returning MPA graduate. [file 12960_2022_781_MOESM4_ESM.docx]

***Additional file 4: Consequence Modelling and Modelled Revenue Scenarios***

The following areas of increased efficiencies and potential gross revenues associated with consequences of MPA graduates returning to practices and having non-clinical administrative tasks covered by a receptionist for half a day, to allow them to undertake higher level duties were considered and are detailed below.

1. ***Consequences from MPA Program for General Practices***

***a) Increase in revenue from potential additional patients seen at the practice***

Increase in revenue for additional patients were conservatively costed at the Australian Medical Benefit Scheme (Item 23) for a basic consult with a general practitioner valued at AUD $38.75 (May 2021) per patient, per visit. This schedule fee is the amount the Australian Government considers appropriate for a standard GP consult. Patients can claim 100% of this fee as a rebate from the Australian government. General practices can charge above the scheduled fee and these patient out-of-pocket costs were excluded from the analysis. This figure was multiplied by the number of potential extra patients per week. The survey results showed a range between 0-12 extra patients per week per practice. This was then extrapolated out to a potential yearly increase in gross revenue of between $0 and $24,180 (Table 4a).

***b1) Modelled increase in revenue from nurses being able to potentially focus on additional higher-revenue activities while MPAs take on their lower order clinical and administrative tasks.***

These activities include performing Health Assessments (MBS Item 703), developing General Practice Management Plans (MBS Item 721) and Team Care Arrangements (MBS Item 723) which can be billed separately, over and above a standard consult. It was conservatively estimated that the Practice Nurse could perform a minimum of two Health Assessments and two Management Plans and a maximum of three of each in a four-hour period, allowing for short breaks between patients. The costing reflects this and is based on the gross additional revenue, not accounting for the cost of relieving the MPA for half a day and their additional wage. Total gross annual revenue could potentially be between $136,474 and $152,672 (mid-point = $144,573) (Table 4 (b1)).

OR

***b2) Modelled increase in revenue from using their clinical skills to offer a greater volume of services and generate more gross revenue.***

In this scenario, the MPA is not substituting for the practice nurse. MPAs and Practice Managers who completed the survey reported that practices were getting more demand for certain services (e.g. ECGs) due to having an MPA onboard. This was confirmed by PHN staff. MPAs were also able to offer more services like a weekly Diabetic Clinic to help patients better manage their condition, leading to better patient care. The extra patients being seen per week also generate more tests and follow-up. In larger group practices, with several GPs generating activity such as tests and investigations, the MPA could also reduce waiting times for those required tests.

These tests include spirometry, audiometry, pregnancy tests, electrocardiograms, and ankle brachial index tests. The increases in gross revenue were conservatively modelled based on reasonable assumptions about the potential frequency of such testing. Increased revenue was based on:

(i) the relevant MBS item numbers for those tests (11505, 11306, 73806, 11707, 11601) ranging from AUD$10.15 to $66.30

Total gross annual revenue could potentially be between $22,332 and $137,904 (Table 4 (b2)).

**c) *Reduced net operational labour costs from being able to use the MPA to backfill 50% of the practice nurses’ duties on a leave day.***

These savings were calculated based on the difference in the wages for a casual registered nurse for the whole day versus the wage of a casual registered nurse for half the day and a receptionist wage for half a day to backfill the MPA. This worked out to a cost-saving of $117 dollars per day or $4,080 per year when applied to 20 days of annual leave and 15 days of personal leave for the nurse as per their entitlements under the NSW Nurses and Midwives Award (Table 4 (c)).

1. ***Modelled Net Revenue Scenarios***

The following scenarios are modelled on potential consequences previously described and include both gross and net revenues.

1. **Modelled Scenario A**

Additional annual gross revenue between $136,474 and $152,672 or a mid -point of $144, 673 may be expected if the PN utilises their 4-hour daily relief from lower order billable and non-billable activities to conduct higher-order billable activities. These could include two extra patient health assessments, either with a GP management plan or a TCA Team Care arrangement. If combined with an expected $12,090 from extra patients, this would give a practice an average gross revenue of $156,663 per annum. Less the expected economic costs of $69,756, the net revenue would be $86,907 a year after graduation (Table 5, Modelled Scenario A).

1. **Modelled Scenario B**

Additional annual gross revenue between $22,256 (if undertaking 2 spirometry tests per day) and $137,904 per annum (if undertaking 8 ankle brachial index tests per day) if the MPA allocates 2 hours a day towards conducting extra billable services (mid-point = $68,952). Combined with $12,090 from extra patients, a gross revenue of $81,042 would be realised by a practice in the 12 months post-graduation. Less the expected economic costs of $69,756, the net revenue during the first post-graduation year would be $11,286 (Table 5, Modelled Scenario B).

1. **Modelled Scenario C**

If able to negotiate a gross pay increase of $3.00 per hour, MPA graduates will have an income increase of $5,948 per annum (less income tax); an increase in pay of $1.50 an hour, will increase annual incomes by $2,974. Given their MPA related economic costs are $3,729, they can expect to recoup their full investment within two years of graduation (Table 5, Modelled Scenario C).

***Examples of Non-Billable Clinical and Administrative tasks within remit of MPA Role Qualifications***

- 1. **Non-billable clinical tasks**
- Application of splints and slings
- Assist with minor procedures
- Assisting with nebulising
- Assisting with plastering and removal of plaster
- Chaperoning
- Infection control measures
- Observations
- Preparation of General Practice Management Plan/Team care Arrangement
- Provide advanced first aid
- Manage first aid services and resources
- Removal of suturing
- Simple wound dressings
- Sterilising
- Triaging
- Urinalysis
- Collecting patient data for required for quality improvement

1. **Non-billable administrative tasks- requiring MPA level clinical** **knowledge**

- Patient recalls and reminders
- Data cleansing
- Extracting financial and clinical data using PenCAT (a commercial clinical audit tool used in general practice)
- 10A reports for Immunisation reminders
- Typing medical reports
- Maintain Work Health and Safety protocols
- Contribution to accreditation – policies and procedures
- Co-ordinate vaccine clinics
- Co-ordination of care – arranging pathology, scheduling of appointments, reminders, allied health appointments
- Cold chain monitoring
- Inventory control
- Maintain medication stocks
- Stock orders including vaccines
- Liaison with My Aged Care (Australian Government-funded aged care portal)
