# Supplementary material for: Impact assessment of the medical practice assisting (MPA) program in general practice in the hunter New England and central coast regions of Australia
Source: Hum Resour Health. 2022 Dec 5;20:81. doi: 10.1186/s12960-022-00781-6 (PMC9721062; doi:10.1186/s12960-022-00781-6)
Supplement: Supplementary file 5 — Additional file 5: Recommendations for optimising the benefits and impacts from the HNECC PHN Medical Practise Assisting Program. Description: Outline of key recommendations for optimising the benefits and impacts from the HNECC PHN Medical Practise Assisting Program as demonstrated through the FAIT Impact assessment. [file 12960_2022_781_MOESM5_ESM.docx]

**Additional file 5-** **Key recommendations**

Key recommendations for better recognition of the MPA Program, reduced variation in the utilisation and remuneration of MPA graduates and greater impacts and benefits for participating practices:

- **Review the content of MPA course to assess if it should be eligible for upgrade to a Diploma level qualification**

***Who?:*** University of New England Partnerships

***Anticipated outcome***: Greater parity with other courses and better recognition by professional organisations like Australian Health Practitioner Regulation Authority (Ahpra)

- **Increase pre-program engagement including effective screening of potential MPA students to ensure suitability and increase potential for completion**

***Who?:*** Primary Health Network

***Anticipated outcome***: Optimise completions and reduce completion time of the course

- **Provide greater direction to General Practices on how to manage the MPA role within the practice team (PHN) and greater planning for integration of the MPA into practices post-graduation ***

***Who?:*** Primary Health Network, participating general practices, MPA graduates, and other practice staff

***Anticipated outcome***: Optimise utilisation of MPAs, reduce burden on practice managers and GPs and increase potential revenue for the practice

** This could include the PHN actively supporting practices to re-engage with the MPA, identify practical and reasonable task redistribution including communication with all staff, potentially restructuring practice facilities, engaging part-time administrative staff, introducing set days and times for specialised clinics and quality assurance. It could also involve the development of templates, resources and exemplar case studies, practical models and utilization of skills, time and space to maximise quality of patient care and productivity*

- **Advocate for greater recognition and promotion of the MPA role including adequate renumeration, a recognised award under** **Australian Health Practitioners Regulation Authority (Ahpra) and acknowledgement of MPA’s increased skills and value**

***Who?:*** Primary Health Network and participating general practices

***Anticipated outcome***: Greater interest in participating in the program by General Practices and their administration staff
